# Neonatal Advanced Practice Nursing: Lessons From American Academy of Pediatrics Clinical Report to the Global South

**DOI:** 10.1002/pdi3.70057

**Published:** 2026-06-17

**Authors:** Aditya Hemendra Bhatt, Somashekhar Marutirao Nimbalkar

**Affiliations:** ^1^ Department of Neonatology, Pramukhswami Medical College, Shree Krishna Hospital Bhaikaka University Anand Gujarat India

**Keywords:** AAP clinical report, lessons to the global south, neonatal nursing

## 1 Introduction

Advanced Practice Registered Nurses (APRNs) have long underpinned neonatal intensive care units (NICUs), with the Neonatal Clinical Nurse Specialist (NCNS) and Neonatal Nurse Practitioner–Board Certified (NNP‐BC) historically anchoring care delivery across newborn and special care nurseries and across all NICU levels. Recent workforce pressures—resident duty‐hour limits, rising NICU admissions despite declining births, and accumulated overtime contributing to burnout—have catalyzed the broader integration of APRN roles, including pediatric acute/primary care nurse practitioners (NPs) and family NPs, necessitating clearer scopes of practice and credentialing to ensure safe neonatal deployment. The American Academy of Pediatrics (AAP) emphasizes the Licensure, Accreditation, Certification, and Education (LACE) alignment to ensure that training, certification, and licensure align with the neonatal populations actually served, particularly at higher‐acuity NICU levels [1]. Globally, low‐ and middle‐income countries (LMICs) face parallel shortages; the WHO roadmap on human resource strategies to improve newborn care highlights the need for skilled cadres, competency‐based education, supportive supervision, and enabling environments as part of human resources for health (HRH) strengthening [2]. India's emerging Nurse Practitioner in Neonatal Nursing (NPNeoN) residency and national dialogues on neonatal nursing capacity illustrate pragmatic LMIC pathways to elevate advanced practice roles in newborn care [3].

## 2 Methods

This narrative synthesis integrates the AAP 2025 clinical report on advanced practice nursing in neonatology with global and LMIC‐focused guidance to contextualize role scope, training, credentialing, and system supports across settings with different income levels. Sources included the AAP report (scope delineation, role definitions, competencies, supervision, and recommendations); WHO's human resource strategies for newborn care in LMICs (cadre recognition, staffing, training pathways, and enabling environments); an integrative review of APRN roles in low‐ and lower‐middle‐income countries (education, regulation, and practice variability); and India‐specific scholarship and policy artifacts on neonatal nursing reform and NNP pathways. The analysis maps LACE‐consistent role preparation to NICU‐level requirements. It examines where role adaptations (e.g., Certified Pediatric Nurse Practitioner–Acute Care [CPNP‐AC], Certified Pediatric Nurse Practitioner–Primary Care [CPNP‐PC], and Family Nurse Practitioner [FNP]) can safely complement NNP‐BCs and NCNSs under structured credentialing and collaboration frameworks [1, 4].

## 3 Results

The NNP‐BC remains the neonatal generalist‐specialist, with graduate preparation, plus neonatal‐population‐specific didactic and supervised NICU clinical hours, and procedural training (e.g., intubation, umbilical access, chest tubes, exchange transfusion, and assisted ventilation), positioning this role for comprehensive care from the delivery room to the high‐acuity NICU [1, 5]. NCNSs complete master/doctor training focused on systems leadership, evidence‐based practice, quality improvement (QI), and staff development, serving as essential practice integrators across neonatal settings [1, 6]. CPNP‐AC certification requires a minimum of 500 supervised direct‐care clinical practice hours in acute care pediatrics (Pediatric Nursing Certification Board strongly recommends 600); however, neonatal‐specific content and NICU rotations are not uniformly required, obligating employers to verify neonatal competencies before NICU deployment [1, 7]. CPNP‐PC certification requires a minimum of 500 supervised direct‐care clinical practice hours in primary care pediatrics, typically in outpatient settings [8], whereas FNP programs include at least 500 faculty‐supervised clinical hours distributed across the lifespan, with comparatively less focus on pediatrics [9]. For all non‐neonatal APRN roles, including FNPs, supervision/collaboration norms vary by jurisdiction; however, neonatal deployment should require neonatology‐specific continuing education, competency verification, and regular credential review [10]. The report concludes that pediatric acute/primary care and FNP roles can complement the capacity of NNP‐BCs and NCNSs for late preterm, low‐acuity, and chronic care populations, freeing NNP‐BCs for the sickest and most premature infants under robust LACE‐aligned credentialing [1].

## 4 Discussion

NICUs already face significant workforce constraints. Global reviews of task‐shifting stress that reassigning duties (e.g., to nurses or NPs) requires clear protocols, ongoing supervision, and clarity of roles to avoid burnout or unsafe practice. Key barriers include limited clinical training sites and preceptors, as well as high patient‐to‐preceptor ratios, which can hamper the expansion of APRN programs. Addressing these barriers will require institutional support—for example, protected training time for preceptors and moderation of patient loads during new APRN orientation.

*Competency validation:* High‐resource settings use structured competency checklists and simulations to ensure readiness. NANN's Competencies & Orientation Toolkit defines core competencies for NNPs (providing care from birth to 2 years) and offers evaluation frameworks. Hospitals often require new NNPs/NCNSs to complete formal orientation, including milestone assessments (e.g., procedural skill labs, written exams, and proctored clinical cases) [11]. LMIC programs could adopt analogous mechanisms, such as national certification exams, portfolio reviews, and standardized competency checklists used during NICU orientation and privileging. For example, a country might adapt NANN's orientation competencies or WHO job aids to the local context, and require periodic skill validation (such as annual stethoscope exams, or resuscitation drills) to maintain privileges.

*Role clarity and collaboration:* It is essential to define each APRN's scope of practice based on their training [1]. National Association of Neonatal Nurses (NANN) emphasizes that providers practicing in NICUs should complete neonatal population‐specific education and achieve neonatal population‐specific national certification [6]. Thus, family or general pediatric NPs should not independently manage unstable or extremely preterm infants. Instead, their scope should be limited (e.g., late‐preterm stabilization, care of convalescing infants, and outpatient follow‐up) and always practiced within a collaborative team. This framework replaces the notion of “physician oversight” with collaborative care models: NICU APRNs should work within an interprofessional team jointly led by neonatology physicians and senior nursing leadership. NANN also recommends that staffing and finance principles be shared with frontline nurses and that nurses participate in developing staffing policies, supporting shared accountability and multidisciplinary care [12]. This aligns with WHO's call for explicit scopes of practice for all cadres and enabling environments that allow practitioners to work to the highest scope permitted [2].

*Global adaptation* *:* In LMIC settings, expansion of the APRN role must be cautious and phased. WHO suggests starting by upgrading existing cadres through short‐term training (3–12 months) and certification before creating entirely new specialty NP cadres. Early pilots (as in India) show promise when new roles are introduced in Level III centers with strong mentorship and monitoring. National policy can standardize APRN scopes: For instance, by defining competencies for neonatal NPs in nursing regulations and linking them to university programs. Continuous quality monitoring is key; countries should embed APRN outcomes in routine newborn care audits (e.g., by tracking neonatal mortality by staffing models).

### 4.1 Policy and Implementation Implications

To implement this model, policymakers should recognize neonatal APRNs as regulated cadres and align education with certification and licensure. Key actions include: Defining national neonatal nursing curricula (with didactic and clinical‐hour minimums); establishing a tiered APRN staffing policy (e.g., NNP/NCNS at high‐acuity centers and CPNP/FNP in step‐down units); and mandating protected NICU posts for APRNs to prevent rotation away from newborn care. Governments and institutions should fund faculty development and residencies to overcome preceptor shortages. As in high‐income countries, APRN productivity should be tied to quality metrics; establishing frameworks such as the National Certification Corporation (NCC) exam or regional certification can help ensure consistency. Collaborative bodies—including nursing councils, pediatric associations, and international partners—can adapt existing tools, for example by translating NANN competency guidelines or WHO training modules for local use. Importantly, any APRN deployment should be accompanied by interprofessional training (e.g., joint simulation) to foster team practice.

*Comparative APRN roles (by training and scope):* Table 1 illustrates how education, clinical hours, and procedures align with each APRN role's intended practice population.

**TABLE 1:** Comparison of APRN roles in neonatal care by training, scope, and competency requirements (adapted from references [1, 13]).

APRN role	Education	Neonatal training (didactic/clinical)	Scope of practice	Key skills/procedures
NNP‐BC	MSN/DNP (neonatal NP); NCC certified	≥ 200 h neonatal didactic; ≥ 750 h NICU	Preterm neonates through age 2 years; all NICU levels	Intubation, ventilation, umbilical/CVC access, chest tubes, exchange transfusion
NCNS	MSN/PhD (neonatal CNS)	MSN/doctoral focus on systems (no set hours)	Quality improvement, staff mentoring	Program development, consultations, no procedures required
CPNP‐AC	MSN/DNP (pediatric acute NP); NCC certified	≥ 750 h pediatric acute care; neonatal content variable	Acutely ill children; with orientation can manage stable infants ≥ 35 weeks	Airway management, vascular access, chest tubes (per local privileging)
CPNP‐PC	MSN/DNP (pediatric primary NP); NCC certified	Education is pediatric‐focused, and program materials commonly define the population focus as newborn to 21 years of age	Outpatient pediatrics; stable term/late‐preterm infants in follow‐up	None (no neonatal procedures required)
FNP	MSN/DNP (family NP); NCC certified	FNP programs include comparatively fewer required pediatric clinical hours because they prepare for lifespan care.	Family primary care; healthy term infants and routine well‐baby care	None; neonatal procedures not required

Abbreviations: APRN, Advanced Practice Registered Nurse; CPNP‐AC, Certified Pediatric Nurse Practitioner–Acute Care; CPNP‐PC, Certified Pediatric Nurse Practitioner–Primary Care; FNP, Family Nurse Practitioner; NCC, National Certification Corporation; NCNS, Neonatal Clinical Nurse Specialist; NNP‐BC, Neonatal Nurse Practitioner–Board Certified; PNCB, Pediatric Nursing Certification Board; QI, quality improvement.

*Team‐based framing:* Throughout these policies, language must emphasize collaboration and shared leadership. For example, APRNs should be described as part of a “neonatal care team” rather than under “physician oversight.” NANN recommends that staffing and finance principles be shared with frontline nurses and that nurses participate in developing staffing policies [12]. Similarly, highlighting patient‐centered teams—including APRNs as key members—aligns with global movements toward interprofessional care.

### 4.2 Scope in LMICs

WHO notes that neonatal nursing is often not recognized or regulated as a specialty in LMICs, with many nurses trained on the job, rotated away from newborn units, and constrained by shortages of equipment, protocols, and supportive infrastructure—conditions that impede quality care for small and sick newborns [2]. The global roadmap recommends standardized competency‐based education, the creation or upgrading of specialized neonatal nursing cadres, effective staffing ratios, protected neonatal postings, and enabling environments (oxygen, phototherapy, continuous positive airway pressure, infection prevention, referral, and transport), all of which parallel the LACE‐aligned rigor underpinning APRN scope in high‐income countries. Evidence from LMICs supports task sharing with clear protocols, mentorship, and limited formularies to extend neonatal care, provided regulation, supervision, and continuing education are in place to safeguard outcomes and professional roles [13, 14].

### 4.3 India as an LMIC Exemplar

India's neonatal nursing capacity is expanding through targeted education, simulation, and continuing professional development; however, uneven standards, faculty shortages, variable NICU exposure, and rural–urban disparities persist, challenging consistency of quality at the point of care. The Indian Nursing Council’s Nurse Practitioner in Neonatal Nursing (NPNeoN) residency reflects a pivotal step toward formalizing advanced neonatal nursing, aligning curricula with specialized competencies, and creating a pathway akin to NNP roles contextualized to national needs [15]. Implementing NNP‐like roles within India's public neonatal network can be phased by pairing high‐acuity hubs with structured mentorship, tele‐education, and simulation while maintaining physician collaboration and local credentialing to ensure safe scope expansion [3, 6]. Creation or expansion of neonatal APRN‐type roles should be phased so that role expansion does not outpace the regulatory structures needed to standardize education, define scope‐of‐practice boundaries, and ensure reliable competency validation through formal credentialing/certification systems, consistent with the WHO's roadmap on newborn‐care human resources [2]. To protect patients and sustain public trust as these roles develop, continuing competency should be embedded as an ongoing safeguard rather than treating certification as a one‐time endpoint; for example, NCC credentials are time‐limited and require periodic maintenance through continuing education and competency assessment [16]. Beyond stating prerequisites for applying the staffing matrix in LMICs, policymakers can organize implementation by convening a national stakeholder taskforce (regulators/nursing councils, neonatology leadership, and universities), mapping workforce supply and NICU acuity needs, defining facility‐level scopes with minimum neonatal didactic/clinical standards, and piloting the model at selected referral centers with structured supervision/mentorship and outcome monitoring.

### 4.4 Staged Implementation Roadmap for LMICs

LMIC adoption of APRN‐like neonatal roles benefits from a staged approach: Define national neonatal competencies, recognize specialized cadres, mandate protected postings, and embed recurrent skills refreshers with audit and QI cycles linked to outcomes and staffing norms. An LMIC‐appropriate advanced practice ladder—building from enhanced neonatal nurse training toward regulated neonatal NP roles—should proceed with regulatory clarity, university partnerships, and financing for faculty development and clinical preceptorships to avoid overextension and ensure competence. India and peers can leverage WHO strategies, national nursing councils, and academic–health system collaboratives to align education, regulation, and service delivery so that scope expansion translates into measurable gains in neonatal survival, quality of care, continuity of care, and workforce sustainability [17].

## 5 Conclusion

This re‐analysis supports a tiered neonatal APRN model centered on NNP‐BC/NCNS leadership for high‐acuity care, complemented by pediatric acute/primary care and FNPs in clearly defined, LACE‐aligned scopes, with neonatologist oversight in intensive settings and rigorous competency maintenance. Extending this framework to LMICs requires formal recognition of neonatal nursing, competency‐based education, protected staffing, supportive environments, and the phased introduction of advanced roles within national regulatory and financing architectures to improve equitable access and quality of care for small and sick newborns.

## Author Contributions

**Aditya Hemendra Bhatt:** conceptualized and designed the study, undertook data collection, and reviewed and revised the manuscript. **Somashekhar Marutirao Nimbalkar:** coordinated and supervised the analysis, and reviewed and revised the manuscript. All authors approved the final manuscript before submission and agree to be accountable for all aspects of the work.

## Funding

The authors have nothing to report.

## Conflicts of Interest

The authors declare no conflicts of interest.

## Data Availability

Data sharing not applicable to this article as no datasets were generated or analyzed during the current study.
